# Supplementary material for: A Multicenter, Open-Label, Phase I/II Study of FN-1501 in Patients with Advanced Solid Tumors
Source: Cancers (Basel). 2023 Apr 29;15(9):2553. doi: 10.3390/cancers15092553 (PMC10177510; doi:10.3390/cancers15092553)
Supplement: Supplementary file 1 [file cancers-15-02553-s001.zip › cancers-2284770-supplementary.pdf]

cancers

MDPI

Supplementary Material: A Multicenter, Open-Label, Phase I/II Study of FN-1501 in Patients with Advanced Solid Tumors

Table S1. Severe Treatment-Emergent Adverse Events (Grade ≥ 3) (Safety Population)

System Organ Class Preferred Term	FN-1501 dose (mg/day), n (%)													
	**2.5 N = 4**	**5 N = 3**	**10 N = 5**	**15 N = 3**	**22.5 N = 4**	**30 N = 3**	**40 N = 4**	**54 N = 3**	**72 N = 3**	**96 N = 5**	**128 N = 3**	**170 N = 4**	**226 N = 4**	**Total N = 48**
Total Patients with at least one TEAE	4 (100)	3 (100)	4 (80)	3 (100)	4 (100)	3 (100)	4 (100)	3 (100)	2 (67)	5 (100)	2 (67)	4 (100)	4 (100)	45 (94)
Total number of TEAEs	20	23	25	41	26	16	21	12	15	33	43	60	46	381
**Blood and lymphatic systemic disorders**														
Anaemia	0	0	0	0	0	0	0	1 (33)	0	0	0	0	0	1 (2)
Iron deficiency anaemia	0	0	0	0	0	0	0	0	0	0	0	0	1 (25)	1 (2)
Neutropenia	0	0	0	0	0	0	0	0	0	0	0	1 (25)	0	1 (2)
Thrombocytopenia	0	0	0	0	0	0	0	0	0	0	0	0	1 (25)	1 (2)
**Endocrine disorders**														
Adrenal insufficiency	0	0	0	0	0	0	0	0	0	0	0	1.(25)		1 (2)
**Gastrointestinal disorders**														
Abdominal distension	0	0	0	0	0	0	1 (25)	0	0	0	0	0	0	1 (2)
Abdominal pain	0	0	0	0	1 (25)	0	0	0	0	0	0	0	0	1 (2)
Diarrhoea	0	1 (33)	0	0	0	0	0	0	0	1 (20)	0	0	1 (25)	3 (6)
Small intestinal obstruction	0	1 (33)	0	0	0	0	0	0	1 (33)	0	0	1 (25)		3 (6)
Stomatitis	0	0	1 (20)	0	0	0	0	0	0	0	0	0	0	1 (2)
**General disorders and administration site conditions**														
Fatigue	1 (25)	0	0	0	0	0	0	1 (33)	0	0	0	0	0	2 (4)
**Infections and infestations**														

**System Organ Class Preferred Term**	**FN-1501 dose (mg/day), n (%)**													
	**2.5 N = 4**	**5 N = 3**	**10 N = 5**	**15 N = 3**	**22.5 N = 4**	**30 N = 3**	**40 N = 4**	**54 N = 3**	**72 N = 3**	**96 N = 5**	**128 N = 3**	**170 N = 4**	**226 N = 4**	**To-tal N = 48**
Bacteraemia	0	0	0	0	0	0	0	0	0		0	1 (25)	0	1 (2)
Bacterial sepsis	0	0	1 (20)	0	0	0	0	0	0	0	0	0	0	1 (2)
Catheter site in-fection	0	0	0	1 (33)	0	0	0	0	0	0	0	0	0	1 (2)
Lung infection	0	0	1 (20)	0	0	0	0	0	0	0	0	0	0	1 (2)
Peritonitis	0	0	0	0	0	0	1 (25)	0	0	0	0	0	0	1 (2)
Pneumonia	0	0	0	0	0	0	0	0	0	1 (20)	0	0	1 (25)	2 (4)
Sepsis	0	0	0	0	0	0	0	0	0	1 (33)	0	0	0	1 (2)
**Injury, poisoning and procedural complications**														
Infusion related reaction	0	0	0	0	0	0	0	0	0	0	0	0	1 (25)	1 (2)
Vascular access complication	0	0	0	1 (33)	0	0	0	0	0	0	0	0	0	1 (2)
**Investigations**														
Aspartate ami-notransferase increased	0	0	0	0	0	0	0	0	0	0	1 (33)	0	0	1 (2)
Blood alkaline phosphatase in-creased	0	0	0	0	0	0	1 (25)	0	0	0	0	0	0	1 (2)
Blood bilirubin increased	0	0	0	0	0	0	1 (25)	0	0	0	0	0	0	1 (2)
Blood creatinine increased	0	0	0	0	0	0	0	0	0	0	1 (33)	0	0	1 (2)
Lipase in-creased	0	0	0	0	0	0	0	0	0	1 (20)	0	0	0	1 (2)
**Metabolism and nutrition disorders**														
Dehydration	0	0	0	0	0	0	0	0	0	0	0	1 (25)	0	1 (2)
Hyponatraemia	0	0	0	0	0	1 (33)	1 (25)	0	0	0	0	1 (25)	1 (25)	4 (8)

System Organ Class Preferred Term	FN-1501 dose (mg/day), n (%)												
	2.5 N = 4	5 N = 3	10 N = 5	15 N = 3	22.5 N = 4	30 N = 3	40 N = 4	54 N = 3	72 N = 3	96 N = 5	128 N = 3	170 N = 4	226 N = 4	To-tal N = 48
Hypophospha-taemia	0	0	0	0	0	0	0	0	0	0	0	0	1 (25)	1 (2)
**Musculoskeletal and connective tissue disorders**														
Back pain	0	0	0	0	0	0	0	0	0	0	0	0	2 (50)	2 (4)
**Nervous system disorders**														
Lethargy	0	0	0	0	0	0	0	0	0	0	0	1 (25)	0	1 (2)
Syncope	0	0	0	0	0	1 (33)	0	0	0	0	0	0	0	1 (2)
**Psychiatric disorders**														
Delirium	0	0	1 (20)	0	0	0	0	0	0	0	0	0	0	1 (2)
**Renal and urinary disorders**														
Acute kidney injury	0	0	0	0	0	0	0	0	0	0	1 (33)	0	0	1 (2)
**Respiratory, thoracic and mediastinal disorders**														
Hypoxia	0	0	0	0	0	0	0	0	0	0	0	1 (25)	0	1 (2)
Pneumonia as-piration	0	0	0	0	0	0	0	0	0	1 (20)	0	0	0	1 (2)
Pneumothorax	0	0	0	0	0	0	0	0	0	1 (20)	0	0	0	1 (2)

Abbreviations: N = number of patients in the dose cohort; n = number of patients in the specified category; TEAE = treatment-emergent adverse event.

Notes: AEs were coded using MedDRA version 20.1. If multiple events with the same PT in a SOC existed for a patient, the most severe event was used. Multiple events in the same SOC for a patient were only counted once for that SOC. The percentage represents the incidence of an event by the SOC or the PT as a percentage of the total number of patients in the treatment group.

Table S2. List of pharmacokinetic parameters evaluated and their definitions

Parameters	Description
AUC_{∞}	The area under the concentration-time curve from time 0 extrapolated to infinity based on the last observed concentration
AUC_{last}	AUC from the time of dosing to the last measurable positive concentration
$t_{1/2}$	Apparent terminal elimination half-life time
MRT_{∞}	Mean residence time extrapolated to infinity base on the last observed concentration
C_{max}	Maximum observed concentration obtained directly from the concentration-time data
T_{max}	Time of maximum observed concentration
CL	Apparent clearance after extravascular administration
Vss	Apparent volume of distribution at steady state
Vz	Apparent volume of distribution during terminal elimination phase

Table S3. Summary of Pharmacokinetic Parameters for FN-1501 (PK Population)

Dose Level	Statistic	AUC_{∞} (h*ng/mL)	AUC_{last} (h*ng/mL)	$t_{1/2}$ (h)	MRT_{∞} (h)	C_{max} (ng/mL)	t_{max} (h)	CL (L/h)	Vss (L)	Vz (L)
2.5 mg	N	4	4	4	4	4	4	4	4	4
	Mean	32.4	29.4	12.8	11.7	12.0	1.2	85.1	913	1510
	Geometric mean	30.7	27.8	12.5	11.0	11.9	1.2	81.4	895	1460
	SD	13.1	11.9	3.50	4.59	1.42	0.10	26.8	206	421
	Geometric CV%	0.376	0.382	0.285	0.440	0.124	0.084	0.376	0.240	0.298
	Median	27.6	25.4	12.7	11.7	12.3	1.1	91.8	937	1520
	Minimum	23.1	20.2	9.04	6.50	10.0	1.1	48.7	664	1020
	Maximum	51.4	46.5	16.8	17.0	13.4	1.3	108	1110	1980
5 mg	N	3	3	3	3	3	3	3	3	3
	Mean	56.5	52.1	18.6	13.7	25.5	1.1	93.9	1320	2570
	Geometric mean	54.8	50.2	18.5	13.3	21.9	1.1	91.3	1220	2440
	SD	18.2	18.2	2.34	3.74	17.3	0.029	25.5	619	932
	Geometric CV%	0.308	0.335	0.127	0.267	0.760	0.026	0.308	0.555	0.435

Dose Level	Statistic	AUC_∞ (h*ng/mL)	AUC_{last} (h*ng/mL)	$t_{1/2}$ (h)	MRT_∞ (h)	C_{max} (ng/mL)	t_{max} (h)	CL (L/h)	Vss (L)	Vz (L)
	Median	46.3	42.3	18.7	12.3	19.7	1.1	108	1340	2940
	Minimum	45.8	40.9	16.3	10.8	11.8	1.1	64.5	696	1520
	Maximum	77.5	73.1	21.0	17.9	45.0	1.1	109	1930	3270
10 mg	N	3	4	3	3	4	4	3	3	3
	Mean	129	119	13.7	12.5	36.9	1.2	84.7	1110	1720
	Geometric mean	124	115	13.5	12.3	33.9	1.2	81.0	998	1580
	SD	41.3	32.6	2.68	2.84	17.5	0.15	32.4	664	925
	Geometric CV%	0.371	0.318	0.210	0.232	0.513	0.13	0.371	0.595	0.533
	Median	142	125	14.5	12.5	34.2	1.1	70.3	773	1300
	Minimum	82.1	73.8	10.7	9.75	20.3	1.0	62.0	685	1080
	Maximum	161	152	15.8	15.4	59.0	1.4	122	1880	2780
15 mg	N	3	3	3	3	3	3	3	3	3
	Mean	153	138	16.6	16.1	35.6	1.2	98.5	1600	2370
	Geometric mean	152	138	16.4	16.0	35.6	1.2	98.4	1570	2320
	SD	8.45	10.8	3.00	2.64	2.15	0.14	5.29	331	536
	Geometric CV%	0.0546	0.0768	0.190	0.165	0.0604	0.11	0.0546	0.215	0.244
	Median	148	134	17.1	16.0	35.6	1.3	101	1620	2510
	Minimum	148	130	13.3	13.6	33.5	1.1	92.4	1260	1770
	Maximum	162	151	19.2	18.9	37.8	1.3	102	1920	2820
22.5 mg	N	2	4	2	2	4	4	2	2	2
	Mean	239	251	18.3	16.4	65.8	1.2	96.9	1600	2570
	Geometric mean	235	241	18.3	16.3	62.2	1.2	95.5	1560	2530
	SD	55.4	87.8	0.494	1.01	24.5	0.19	22.5	466	663
	Geometric CV%	0.237	0.338	0.027	0.0617	0.405	0.16	0.237	0.302	0.266
	Median	239	227	18.3	16.4	65.0	1.2	96.9	1600	2570
	Minimum	200	178	18.0	15.7	40.0	1.0	81.0	1270	2100
	Maximum	278	374	18.7	17.1	93.0	1.4	113	1930	3040
30 mg	N	3	3	3	3	3	3	3	3	3

Dose Level	Statistic	AUC_{∞} (h*ng/mL)	AUC_{last} (h*ng/mL)	$t_{1/2}$ (h)	MRT_{∞} (h)	C_{max} (ng/mL)	t_{max} (h)	CL (L/h)	Vss (L)	Vz (L)
	Mean	341	297	12.4	13.4	87.8	1.1	89.1	1130	1500
	Geometric mean	339	294	10.4	11.3	81.6	1.1	88.5	1000	1330
	SD	47.1	55.8	7.43	8.49	37.5	0.11	13.3	619	786
	Geometric CV%	0.145	0.202	0.964	0.892	0.520	0.099	0.145	0.690	0.749
	Median	360	323	14.8	13.5	95.3	1.1	83.2	1120	1780
	Minimum	288	233	4.10	4.89	47.2	1.0	79.9	510	617
	Maximum	376	336	18.4	21.9	121	1.3	104	1750	2120
40 mg	N	4	4	4	4	4	4	4	4	4
	Mean	499	437	18.7	18.3	128	1.1	83.6	1450	2160
	Geometric mean	488	432	18.2	17.6	122	1.1	81.9	1440	2150
	SD	122	77.2	5.68	6.19	41.9	0.044	18.6	185	195
	Geometric CV%	0.238	0.176	0.277	0.313	0.383	0.039	0.238	0.130	0.0911
	Median	472	427	16.2	16.0	132	1.1	85.1	1460	2160
	Minimum	384	356	15.3	13.8	72.7	1.1	59.9	1240	1950
	Maximum	668	538	27.2	27.4	174	1.3	104	1640	2350
54 mg	N	3	3	3	3	3	3	3	3	3
	Mean	868	773	19.6	16.9	323	1.1	63.9	1080	1820
	Geometric mean	856	760	18.9	15.9	320	1.1	63.1	1010	1720
	SD	181	180	6.44	7.34	55.6	0.05	12.4	516	740
	Geometric CV%	0.205	0.224	0.317	0.438	0.183	0.046	0.205	0.467	0.445
	Median	813	674	16.5	14.6	344	1.1	66.5	826	1780
	Minimum	721	663	15.2	11.0	260	1.0	50.4	736	1110
	Maximum	1070	980	27.0	25.2	365	1.1	74.9	1670	2580
72 mg	N	3	3	3	3	3	3	3	3	3
	Mean	823	742	17.2	16.8	161	1.3	94.2	1580	2370
	Geometric mean	792	712	17.2	16.8	152	1.3	90.9	1530	2250
	SD	292	275	1.58	1.12	67.7	0.24	28.8	489	833

Dose Level	Statistic	AUC_{∞} (h*ng/mL)	AUC_{last} (h*ng/mL)	$t_{1/2}$ (h)	MRT_{∞} (h)	C_{max} (ng/mL)	t_{max} (h)	CL (L/h)	Vss (L)	Vz (L)
	Geometric CV%	0.345	0.359	0.0917	0.0656	0.414	0.19	0.345	0.360	0.423
	Median	705	617	17.1	16.4	134	1.4	102	1840	2780
	Minimum	609	552	15.7	16.0	111	1.1	62.3	1020	1410
	Maximum	1160	1060	18.9	18.1	238	1.6	118	1890	2920
96 mg	N	4	5	4	4	5	5	4	4	4
	Mean	1650	1420	16.1	15.4	494	1.1	59.1	921	1410
	Geometric mean	1640	1410	14.9	14.0	431	1.1	58.7	822	1260
	SD	229	236	6.28	6.58	289	0.13	8.20	425	682
	Geometric CV%	0.140	0.165	0.522	0.580	0.638	0.12	0.140	0.663	0.652
	Median	1640	1330	17.7	17.3	489	1.1	59.2	992	1510
	Minimum	1440	1190	7.29	6.50	236	1.0	51.3	343	555
	Maximum	1870	1710	21.7	20.4	944	1.3	66.7	1360	2090
128 mg	N	3	3	3	3	3	3	3	3	3
	Mean	1980	1730	17.8	18.1	533	1.1	79.9	1320	1790
	Geometric mean	1780	1590	17.0	17.6	460	1.1	72.1	1270	1770
	SD	1120	850	7.00	5.53	344	0.11	42.1	473	366
	Geometric CV%	0.620	0.531	0.391	0.292	0.757	0.095	0.620	0.349	0.203
	Median	1680	1550	15.5	15.0	425	1.1	76.2	1130	1710
	Minimum	1030	980	12.3	14.8	237	1.1	39.7	972	1470
	Maximum	3220	2650	25.7	24.5	910	1.3	124	1860	2190
170 mg	N	4	4	4	4	4	4	4	4	4
	Mean	3760	3340	15.0	15.8	955	1.3	56.5	757	1120
	Geometric mean	3370	3080	14.6	14.6	910	1.3	50.4	735	1060
	SD	1980	1500	3.64	7.89	330	0.26	31.5	218	419
	Geometric CV%	0.595	0.515	0.253	0.454	0.375	0.20	0.595	0.284	0.397
	Median	3460	3250	15.0	12.5	972	1.2	49.5	715	1080
	Minimum	1690	1640	11.1	10.5	606	1.1	26.4	538	713
	Maximum	6440	5230	18.7	27.5	1270	1.6	101	1060	1600

Dose Level	Statistic	AUC∞ (h*ng/mL)	AUClast (h*ng/mL)	t1/2 (h)	MRT∞ (h)	Cmax (ng/mL)	tmax (h)	CL (L/h)	Vss (L)	Vz (L)
226 mg	N	4	4	4	4	4	4	4	4	4
	Mean	6070	5620	14.2	15.2	1180	1.2	39.4	569	787
	Geometric mean	5900	5490	14.1	14.8	1160	1.2	38.3	568	778
	SD	1670	1390	2.20	3.92	232	0.12	10.7	30.8	139
	Geometric CV%	0.284	0.255	0.147	0.258	0.188	0.10	0.284	0.0558	0.184
	Median	5950	5600	13.3	14.4	1110	1.2	39.8	583	805
	Minimum	4600	4350	12.7	11.9	979	1.0	29.0	522	619
	Maximum	7790	6920	17.4	20.1	1510	1.3	49.2	586	921

CV% = arithmetic percent coefficient of variation; N =number of patients with data; SD = standard deviation.

Table S4. Summary of Pharmacokinetic Parameters for M3 (PK Population)

Dose Level	Statistic	AUC∞ (h*ng/mL)	AUClast (h*ng/mL)	t1/2 (h)	Cmax (ng/mL)	tmax (h)
2.5 mg	N	0	2	1	2	2
	Mean	-	0.234	1.43	0.172	1.2
	Geometric mean	-	0.234	1.43	0.171	1.2
	SD	-	0.0185	NC	0.0212	0.12
	Geometric CV%	-	0.0793	NC	0.124	0.097
	Median	-	0.234	1.43	0.172	1.2
	Minimum	-	0.221	1.43	0.157	1.1
	Maximum	-	0.247	1.43	0.187	1.3
5 mg	N	0	2	0	2	2
	Mean	-	0.364	-	0.172	1.5
	Geometric mean	-	0.360	-	0.169	1.5
	SD	-	0.0778	-	0.0389	NC
	Geometric CV%	-	0.218	-	0.232	NC
	Median	-	0.364	-	0.172	1.5
	Minimum	-	0.309	-	0.144	1.5
	Maximum	-	0.419	-	0.199	1.5
10 mg	N	0	4	1	4	4
	Mean	-	2.67	10.1	0.268	1.8
	Geometric mean	-	1.22	10.1	0.242	1.8

Dose Level	Statistic	AUC$_{\infty}$ (h*ng/mL)	AUC$_{last}$ (h*ng/mL)	$t_{1/2}$ (h)	C_{max} (ng/mL)	t_{max} (h)
	SD	-	3.48	NC	0.141	0.13
	Geometric CV%	-	2.98	NC	0.549	0.073
	Median	-	1.33	10.1	0.232	1.9
	Minimum	-	0.321	10.1	0.153	1.7
	Maximum	-	7.71	10.1	0.456	2.0
15 mg	N	1	3	3	3	3
	Mean	3.80	1.68	7.16	0.308	1.7
	Geometric mean	3.80	1.58	6.97	0.291	1.6
	SD	NC	0.705	2.06	0.133	0.59
	Geometric CV%	NC	0.445	0.299	0.416	0.39
	Median	3.80	1.56	7.04	0.235	1.7
	Minimum	3.80	1.04	5.17	0.227	1.1
	Maximum	3.80	2.44	9.28	0.461	2.3
22.5 mg	N	2	4	4	4	4
	Mean	4.30	3.08	9.27	0.578	1.6
	Geometric mean	4.30	2.96	8.54	0.525	1.6
	SD	0.165	0.905	4.01	0.244	0.51
	Geometric CV%	0.0383	0.337	0.517	0.598	0.32
	Median	4.30	3.20	9.40	0.655	1.5
	Minimum	4.19	1.87	4.49	0.234	1.1
	Maximum	4.42	4.03	13.8	0.769	2.3
30 mg	N	2	3	3	3	3
	Mean	3.86	8.09	19.9	0.516	1.1
	Geometric mean	3.73	4.91	11.2	0.495	1.1
	SD	1.38	9.51	25.2	0.165	0.029
	Geometric CV%	0.378	1.84	2.03	0.372	0.027
	Median	3.86	3.41	5.37	0.583	1.1
	Minimum	2.88	1.82	5.35	0.328	1.0
	Maximum	4.84	19.0	49.1	0.636	1.1
40 mg	N	2	4	4	4	4
	Mean	6.95	4.88	14.7	0.558	1.9
	Geometric mean	6.73	4.70	13.3	0.554	1.8
	SD	2.41	1.54	7.79	0.0789	0.69
	Geometric CV%	0.366	0.333	0.553	0.141	0.38
	Median	6.95	4.85	12.9	0.552	1.8

Dose Level	Statistic	AUC$_{\infty}$ (h*ng/mL)	AUC$_{last}$ (h*ng/mL)	$t_{1/2}$ (h)	C_{max} (ng/mL)	t_{max} (h)
	Minimum	5.24	3.51	7.30	0.474	1.3
	Maximum	8.65	6.31	25.6	0.655	2.7
54 mg	N	3	3	3	3	3
	Mean	21.9	18.0	21.0	1.65	1.8
	Geometric mean	21.1	17.1	20.6	1.35	1.8
	SD	7.45	7.00	5.16	1.35	0.24
	Geometric CV%	0.342	0.405	0.245	0.871	0.14
	Median	19.9	16.8	19.8	0.894	1.8
	Minimum	15.6	11.7	16.5	0.855	1.5
	Maximum	30.1	25.6	26.6	3.21	2.0
72 mg	N	3	3	3	3	3
	Mean	18.3	15.4	16.5	1.84	1.7
	Geometric mean	17.5	14.4	16.3	1.80	1.6
	SD	6.38	5.94	3.45	0.485	0.56
	Geometric CV%	0.410	0.476	0.224	0.275	0.38
	Median	20.5	17.6	17.6	1.84	1.8
	Minimum	11.2	8.60	12.7	1.36	1.1
	Maximum	23.4	19.8	19.3	2.33	2.2
96 mg	N	5	5	5	5	5
	Mean	57.5	42.6	19.3	3.13	1.8
	Geometric mean	40.7	32.8	18.0	2.65	1.8
	SD	60.7	37.6	7.54	1.71	0.29
	Geometric CV%	1.05	0.908	0.442	0.807	0.18
	Median	30.7	25.8	18.1	2.60	1.9
	Minimum	18.0	14.3	9.62	0.854	1.3
	Maximum	164	107	29.7	4.90	2.0
128 mg	N	2	3	2	3	3
	Mean	39.5	26.1	17.5	3.73	1.6
	Geometric mean	39.3	22.6	17.1	2.69	1.6
	SD	5.36	14.3	5.60	3.27	0.35
	Geometric CV%	0.137	0.832	0.334	1.43	0.21
	Median	39.5	32.7	17.5	3.01	1.5
	Minimum	35.7	9.78	13.6	0.890	1.3
	Maximum	43.3	35.9	21.5	7.30	2.0
170 mg	N	4	4	4	4	4

**Dose Level**	**Statistic**	**AUC_{∞} (h*ng/mL)**	**AUC_{last} (h*ng/mL)**	**$t_{1/2}$ (h)**	**C_{max} (ng/mL)**	**t_{max} (h)**
	Mean	9.38	83.7	15.6	8.72	1.4
	Geometric mean	84.8	75.9	15.5	7.48	1.4
	SD	49.0	42.1	2.72	4.68	0.16
	Geometric CV%	0.556	0.550	0.180	0.796	0.11
	Median	83.8	77.0	15.8	9.24	1.5
	Minimum	46.8	41.9	12.3	2.80	1.3
	Maximum	161	139	18.6	13.6	1.6
226 mg	N	4	4	4	4	4
	Mean	185	165	16.5	16.3	1.5
	Geometric mean	168	149	16.3	14.1	1.5
	SD	82.8	76.7	2.55	9.31	0.26
	Geometric CV%	0.603	0.613	0.152	0.733	0.17
	Median	194	169	16.0	15.8	1.4
	Minimum	75.6	67.1	14.0	6.06	1.3
	Maximum	277	254	19.9	27.6	1.9
CV% = arithmetic percent coefficient of variation; N =number of patients with data; NC = Not calculated; SD = standard deviation.						

Table S5. Summary of Best RECIST Response (ITT Population)

**Response**	**FN-1501 dose (mg/day), n (%)**													
	**2.5 N = 4**	**5 N = 3**	**10 N = 5**	**15 N = 3**	**22.5 N = 4**	**30 N = 3**	**40 N = 4**	**54 N = 3**	**72 N = 3**	**96 N = 5**	**128 N = 3**	**170 N = 4**	**226 N = 4**	**Total N = 48**
Complete Response	0	0	0	0	0	0	0	0	0	0	0	0	0	0
Partial Response	0	0	0	0	0	0	1 (25)	0	0	0	0	0	0	1 (2)
Stable Disease	0	0	1 (20)	3 (100)	2 (50)	3 (100)	1 (25)	0	0	2 (40)	1 (33)	2 (50)	0	15 (31)
Progressive Disease	3 (75)	2 (67)	2 (40)	0	2 (50)	0	1 (25)	2 (67)	3 (100)	0	1 (33)	1 (25)	1 (25)	18 (38)
Not Evaluable	0	1 (33)	0	0	0	0	0	0	0	0	0	0	0	1 (2)

Response	FN-1501 dose (mg/day), n (%)													
	**2.5 N = 4**	**5 N = 3**	**10 N = 5**	**15 N = 3**	**22.5 N = 4**	**30 N = 3**	**40 N = 4**	**54 N = 3**	**72 N = 3**	**96 N = 5**	**128 N = 3**	**170 N = 4**	**226 N = 4**	**Total N = 48**
Not Reported	1 (25)	0	2 (40)	0	0	0	1 (25)	1 (33)	0	3 (60)	1 (33)	1 (25)	3 (75)	13 (27)
ORR	0.0%	0.0%	0.0%	0.0%	0.0%	0.0%	25.0%	0.0%	0.0%	0.0%	0.0%	0.0%	0.0%	2.1%
95% CI	0.0, 60.2	0.0, 70.8	0.0, 52.2	0.0, 70.8	0.0, 60.2	0.0, 70.8	0.6, 80.6	0.0, 70.8	0.0, 70.8	0.0, 52.2	0.0, 70.8	0.0, 60.2	0.0, 60.2	0.1, 11.1
DCR	0.0%	0.0%	20.0%	100.0%	50.0%	100.0%	50.0%	0.0%	0.0%	40.0%	33,3%	50.0%	0.0%	33.3%
95% CI	0.0, 60.2	0.0, 70.8	0.5, 71.6	29.2, 100.0	6.8, 93.2	29.2, 100.0	6.8, 93.2	0.0, 70.8	0.0, 70.8	5.3, 85.3	0.8, 90.6	6.8, 93.2	0.0, 60.2	20.4, 48.4

Abbreviations: CI = confidence interval; DCR = disease control rate; N = number of patients in the dose cohort; n = number of patients in the specified category; ORR = objective response rate.

Table S6. Summary of Overall Survival (ITT Population)

	FN-1501 dose (mg/day)													
	**2.5 N = 4**	**5 N = 3**	**10 N = 5**	**15 N = 3**	**22.5 N = 4**	**30 N = 3**	**40 N = 4**	**54 N = 3**	**72 N = 3**	**96 N = 5**	**128 N = 3**	**170 N = 4**	**226 N = 4**	**Total N = 48**
Number of events	2	2	2	0	3	0	1	2	0	2	0	0	1	15

	**FN-1501 dose (mg/day)**													
	**2.5 N = 4**	**5 N = 3**	**10 N = 5**	**15 N = 3**	**22.5 N = 4**	**30 N = 3**	**40 N = 4**	**54 N = 3**	**72 N = 3**	**96 N = 5**	**128 N = 3**	**170 N = 4**	**226 N = 4**	**Total N = 48**
Median (months)	2.0	2.6	NE	NE	5.4	NE	NE	3.2	NE	3.1	NE	NE	NE	8.2
95% CI	0.3, NE	1.8, NE	0.6, NE	NE, NE	2.4, NE	NE, NE	0.8, NE	2.3, NE	NE, NE	1.0, NE	NE, NE	NE, NE	0.8, NE	3.2, NE
**Landmark Progression Free Survival Rate**														
3 months	37.5	50.0	60.0	NA	50.0	NA	75.0	66.7	NA	75.0	NA	NA	66.7	71.7
95% CI	1.1, 80.8	0.6, 91.0	12.6, 88.2	-	5.8, 84.5	-	12.8, 96.1	5.4, 94.5	-	12.8, 96.1	-	-	5.4, 94.5	54.1, 83.5
6 months	37.5	0.0	60.0	NA	50.0	NA	75.0	0.0	NA	37.5	NA	NA	66.7	59.1
95% CI	1.1, 80.8	NE, NE	12.6, 88.2	-	5.8, 84.5	-	12.8, 96.1	NE, NE	-	1.1, 80.8	-	-	5.4, 94.5	39.4, 74.2
9 months	37.5	0.0	60.0	NA	25.0	NA	75.0	0.0	NA	37.5	NA	NA	66.7	44.3
95% CI	1.1, 80.8	NE, NE	12.6, 88.2	-	(0.9, 66.5)	-	12.8, 96.1	NE, NE	-	1.1, 80.8	-	-	5.4, 94.5	16.7, 69.0
12 months	37.5	0.0	60.0	NA	25.0	NA	75.0	0.0	NA	37.5	NA	NA	66.7	44.3
95% CI	1.1, 80.8	NE, NE	12.6, 88.2	-	(0.9, 66.5)	-	12.8, 96.1	NE, NE	-	1.1, 80.8	-	-	5.4, 94.5	16.7, 69.0

Abbreviations: CI = confidence interval; N = number of patients in the dose cohort; NA = not applicable; NE = not evaluable.

Table S7. Summary of Progression Free Survival (ITT Population)

	**FN-1501 dose (mg/day)**													
	**2.5 N = 4**	**5 N = 3**	**10 N = 5**	**15 N = 3**	**22.5 N = 4**	**30 N = 3**	**40 N = 4**	**54 N = 3**	**72 N = 3**	**96 N = 5**	**128 N = 3**	**170 N = 4**	**226 N = 4**	**Total N = 48**
Number of events	4	3	5	3	4	2	3	2	3	3	2	3	2	39
Median (months)	1.8	1.7	1.4	4.1	5.0	3.4	2.6	1.4	1.3	2.7	3.1	2.2	1.1	1.8
95% CI	0.3, NE	1.5, NE	0.6, NE	3.2, NE	1.4, NE	2.7, NE	0.6, NE	1.3, NE	1.2, NE	1.0, NE	0.8, NE	1.3, NE	0.8, NE	1.4, 2.7
**Landmark Progression Free Survival Rate**														
3 months	0.0	0.0	20.0	NA	50.0	50.0	33.3	0.0	0.0	37.5	66.7	33.3	0.0	31.1
95% CI	NE, NE	NE, NE	0.8, 58.2	-	5.8, 84.5	0.6, 91.0	0.9, 77.4	NE, NE	NE, NE	1.1, 80.8	5.4, 94.5	0.9, 77.4	NE, NE	17.6, 45.7
6 months	0.0	0.0	0.0	33.3	50.0	0.0	0.0	0.0	0.0	0.0	0.0	33.3	0.0	11.3
95% CI	NE, NE	NE, NE	NE, NE	0.9, 77.4	5.8, 84.5	NE, NE	NE, NE	NE, NE	NE, NE	NE, NE	NE, NE	0.9, 77.4	NE, NE	3.7, 23.8
9 months	0.0	0.0	0.0	0.0	25.0	0.0	0.0	0.0	0.0	0.0	0.0	33.3	0.0	5.7
95% CI	NE, NE	NE, NE	NE, NE	NE, NE	0.9, 66.5	NE, NE	NE, NE	NE, NE	NE, NE	NE, NE	NE, NE	0.9, 77.4	NE, NE	1.0, 16.5
12 months	0.0	0.0	0.0	0.0	25.0	0.0	0.0	0.0	0.0	0.0	0.0	0.0	0.0	2.8
95% CI	NE, NE	NE, NE	NE, NE	NE, NE	0.9, 66.5	NE, NE	NE, NE	NE, NE	NE, NE	NE, NE	NE, NE	NE, NE	NE, NE	0.2, 12.5

Abbreviations: CI = confidence interval; N = number of patients in the dose cohort; NA = not applicable; NE = not evaluable.
